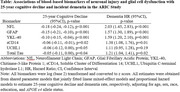# Blood biomarkers of neuronal injury and glial cell dysfunction and 25‐year cognitive decline and incident dementia in the ARIC study

**DOI:** 10.1002/alz70856_104381

**Published:** 2025-12-25

**Authors:** Srishti Shrestha, Xiaoqian Zhu, B Gwen Windham, Kevin J. Sullivan, Priya Palta, Rebecca F. Gottesman, Prashanthi Vemuri, Sudha Seshadri, Thomas H. Mosley, Michael E. Griswold, Myriam Fornage

**Affiliations:** ^1^ University of Mississippi Medical Center, The MIND Center, Jackson, MS, USA; ^2^ University of North Carolina at Chapel Hill, Chapel Hill, NC, USA; ^3^ National Institute of Neurological Disorders & Stroke, Bethesda, MD, USA; ^4^ Department of Radiology, Mayo Clinic, Rochester, MN, USA; ^5^ Glenn Biggs Institute for Alzheimer's & Neurodegenerative Diseases, University of Texas Health Science Center, San Antonio, TX, USA; ^6^ The Brown Foundation Institute of Molecular Medicine, McGovern Medical School, The University of Texas Health Science Center at Houston, Houston, TX, USA

## Abstract

**Background:**

Prospective investigations on blood biomarkers for Alzheimer's disease and related‐dementias (ADRDs) and cognitive impairment provide important insights into their clinical utility in early disease diagnosis and risk stratification, but have been primarily limited to AD pathologies (e.g. amyloid‐beta and hyperphosphorylated‐tau aggregation). Studies of biomarkers reflecting other pathogenic processes that contribute to ADRDs, especially in community‐based cohorts, are lacking. We examined associations of blood biomarkers reflecting glial and neuronal dysfunction and neuroinflammation, including soluble cluster of differentiation‐14 (sCD14), chitinase‐3‐like protein‐1 (YKL‐40), glial‐fibrillary acidic protein (GFAP), neurofilament‐light chain (NfL), total‐Tau, and Ubiquitin C‐terminal hydrolase L1 (UCH‐L1) with cognitive decline and incident dementia in ARIC study participants.

**Methods:**

Blood biomarkers were measured in 1,864 ARIC Visit 3 (1993‐1995) participants using the Meso Scale Discovery assay (YKL‐40), the Quanterix Simoa Neurology 4‐Plex assay (total‐Tau, NfL, GFAP, UCH‐L1), and a commercial ELISA assay (sCD14, R&D Systems); global cognition z‐scores were derived from three cognitive tests (measuring memory, language, and executive function) administered up to six times over 25 years, and incident dementia cases were ascertained via in‐person cognitive assessments (expert‐adjudicated), informant‐based interviews, and hospitalization and death records through 2018. We used shared parameter models to simultaneously fit linear mixed‐effect models and proportional hazard models to estimate 25‐year cognitive decline and dementia rates separately for each biomarker (log‐base2‐transformed), adjusting for covariates (Table).

**Results:**

Among 1,864 participants (mean±SD age:63±4.5 years, 60% female, 49% self‐reported Black), 27% developed dementia. Higher levels of each biomarker were associated with faster cognitive decline. All but UCH‐L1 were associated with elevated dementia risk (Table). For example, one standard deviation (SD) higher log‐base2 YKL‐40 was associated with ‐0.10 SD steeper 25‐year cognitive decline (95%CI:‐0.15,‐0.04) and 59% higher dementia hazard (hazard ratio (HR):1.59 (95%CI:1.20,2.10); likewise, sCD14 was associated with an additional cognitive decline of ‐0.06 (95%CI:‐0.11,‐0.01) and 38% higher dementia hazard (HR:1.38 (95%CI:1.08,1.76)).

**Conclusions:**

Blood biomarkers reflecting neuropathological processes, including microglial activation, astrocytic reactivity, and neuronal degeneration, were associated with faster cognitive decline and increased dementia risk, suggesting that they may aid in risk stratification and intervention strategies for ADRDs. Future analyses will examine whether these associations differ by race and *APOE‐ε4 s*tatus.